# Effect of lateral oblique cyclic loading on microleakage and screw loosening of implants with different connections

**DOI:** 10.15171/joddd.2018.028

**Published:** 2018-09-18

**Authors:** Hakimeh Siadat, Hossain Najafi, Marzieh Alikhasi, Babak Falahi, Elaheh Beyabanaki, Farid Zayeri

**Affiliations:** ^1^ Dental Implant Research Center, Dentistry Research Institute, Tehran University of Medical Sciences, Tehran, Iran; ^2^ Department of Prosthodontics, Faculty of Dentistry, Tehran University of Medical Sciences, Tehran, Iran; ^3^ Dental Research Center, Dentistry Research Institute, Tehran University of Medical Sciences, Tehran, Iran; ^4^ Research Institute for Nuclear Medicine, Shariati Hospital, Tehran University of Medical Sciences, Tehran, Iran; ^5^ Department of Prosthodontics, Faculty of Dentistry, Shahid Beheshti University of Medical Sciences, Tehran, Iran; ^6^ Proteomics Research Center, Shahid Beheshti University of Medical Sciences, Tehran, Iran; ^7^ Department of Biostatics, Faculty of Paramedical Sciences, Shahid Beheshti University of Medical Sciences, Tehran, Iran

**Keywords:** Dental implant‒abutment connection, leakage, gamma rays, torque

## Abstract

***Background.*** The implant connection type might affect microleakage and screw loosening in two-piece implants. The aim of this study was to measure microleakage and screw loosening of two connections of Noble Biocare implant system before and after cyclic loading.

***Methods.*** Twelve samples were categorized into two groups: external hexagon (Branemark) and internal hexagon connection (Noble Active) and two implants as controls. The abutments were tightened to a 35 Ncm torque. Initial torque loss (ITL) was measured five minutes after retightening the abutment, using a digital torque wrench. The samples were covered with putty material to the abutment‒implant junction. Customized metal crowns with 45° inclinations were placed on the abutments and cyclic loading was performed accordingly. Thereafter, the secondary torque loss (STL) was measured. Microleakage test was also performed. Data were analyzed with Mann-Whitney and Wilcoxon tests (α=0.05).

***Results.*** There were no statistically significant differences between the two phases of gamma counting between and within two groups (P>0.05). However, STL after cyclic loading was less than ITL in both groups (P=0.042).

***Conclusion.*** Connection type and cyclic loading had no significant effect on microleakage. Furthermore, the internal connection had less TL as compared to the external connection. In addition, the STLs were less than ITLs in both groups.

## Introduction

Microleakage at the implant‒abutment interface and screw loosening are two major issues in two-piece implant systems.^1^ Screw loosening could result in misfit in the implant‒abutment interface,^2^ leading to several biomechanical complications such as bacterial microleakage, and screw or/and framework fractures.^3^ Bacterial microleakage has also been shown to be related to peri-implantitis and bone loss around the implant.^4^ Screw loosening is generally a result of inadequate or loss of preload following improper initial torquing, screw deformation, screw roughness wear, overloading, and micromovements at the joint due to functional loading.^5^

Generally, there are two types of implant‒abutment connections, including external and internal hex connections. There are basic differences between these two connections in terms of stress dissipation and joint stability. Also, one of the major issues of the two-piece implants is the gap created between two surfaces. The proximity of this micro-gap to the alveolar crest could be the reason for 1 mm of bone loss during the first year of loading.^6^ Therefore, the presence of micro-gap in the implant‒abutment interface is a biomechanical issue, since it is associated with bacterial infiltration and also micromovement and screw loosening.^7^ The stress in the external butt joint connection is mainly transferred to the screw, while in the internal cone connection it is passed on to the internal walls of the implant.^7,8^ There are several factors that have a role in screw loosening phenomenon in two-piece implant systems. These factors include connection geometry design (such as height/depth of anti-rotation, screw design, screw and platform diameter), the amount of applied load, the amount of eccentric loading, height of crown, height of abutment, length of cantilever and the amount of preload.^9-11^

The factors that could affect the amount of bacterial infiltration in the implant–abutment interface include fit accuracy between the components, the amount of preload and micromovements between the jointed parts of the system during loading.^12,13^ Therefore, there has been an attempt to reduce the chance of bacterial infiltration by improving the fit and stability between the connected parts.^14^ According to previous studies the implant connection design could also be a determining factor in the bacterial leakage in different implant systems.^15,16^ In this context, it has been reported that implants with a locking taper connection exhibit more resistance to microleakage^16^ compared to flat-to-flat or tube-in-tube connections.^15^ It has also been suggested that internal conical connection is mechanically more stable, while implants with external hexed connections have a higher chance for instability and leakage.^15^ However, according to Jansen et al^17^ even more internal tight connections such as Morse taper is not completely safe against bacterial leakage. Moreover, it has been shown that screw-connected joints are not thoroughly resistant to fluid seepage and microleakage.^18^ It seems that other options, including one-piece implants or pure interference‒fit connections (locking-taper) are more suitable in terms of eliminating the risk of joint instability and microleakage.^19^

Noble Biocare implant system is one of the pioneers and is the most commonly used dental implant system in the practice of implant dentistry. The aim of the present study was to evaluate and compare the effect of two implant‒abutment connection designs on microleakage and screw loosening before and after cyclic loading. The null hypothesis was that there is no difference between two implant connections in terms of microleakage and screw loosening before and after cyclic loading.

## Methods

**Figure 1:**
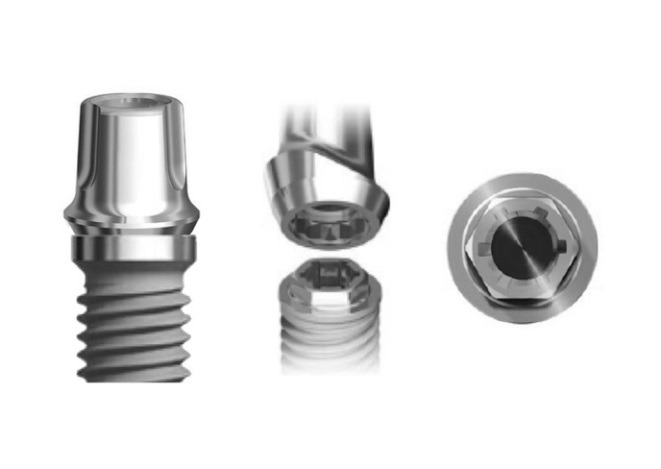
Branemark external hexagon implant (3.75 mm in diameter) and snappy abutment (1 mm in gingival height)

**Figure 2:**
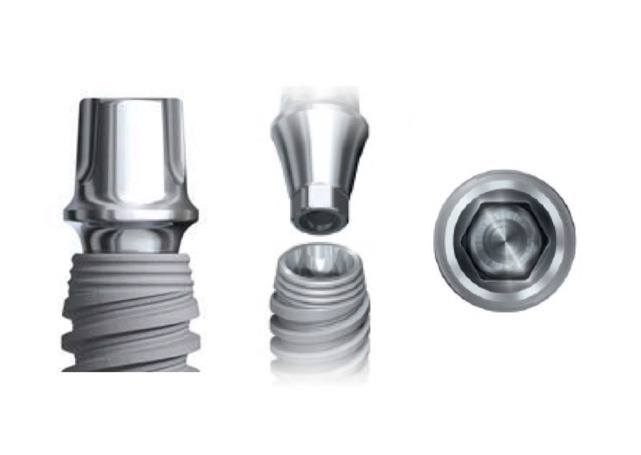
Noble Active conical internal hexagon implant (4.3 mm in diameter) and snappy abutment (1.5 mm in gingival height)

**Figure 3:**
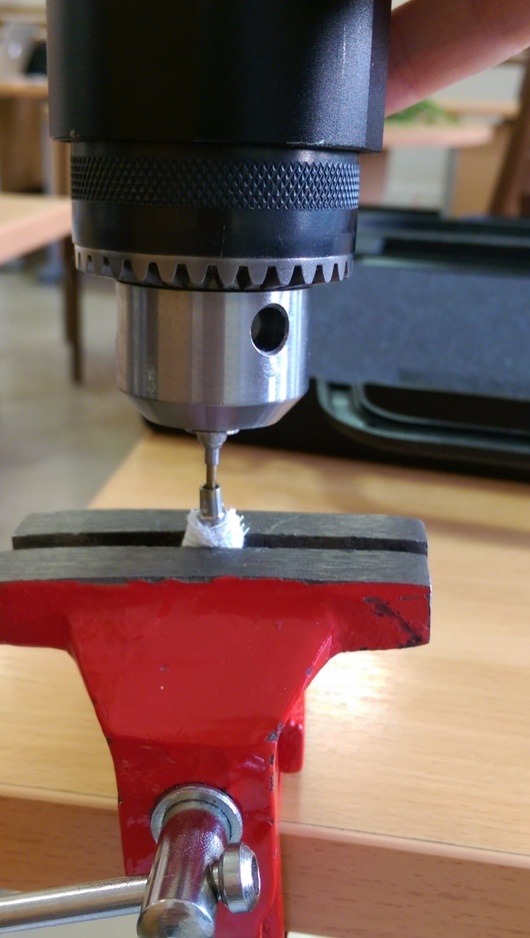
Recording torque value in two implant systems using an electronic torque wrench.

Twelve implants (13 mm in height) were categorized into two groups, with the first group consisting of external hexagon connection (Branemark, Nobel Biocare AB, Göteborg, Sweden) (3.75 mm in diameter) (Figure 1) and the second group consisting of conical internal hexagon connection (Noble Active, Nobel Biocare AB, Göteborg, Sweden) (4.3 mm in diameter) (Figure 2). Two implant‒abutment assemblies were used as negative and positive controls. Snappy abutments (Nobel Biocare, Goteberg, Sweden) with the lowest gingival heights (1 mm for Branemark and 1.5 mm for Nobel Active) were fastened to the implants and torqued to 35 Ncm using an electronic torque controller accurate to 0.1 Ncm (Trinkle Enterprise Co, Taichung, Taiwan). Each sample was mounted in a rigid auto-polymerizing acrylic resin block (Rapid Repair, Meliodent, Heraeus Kulzer GmbH, Germany) to inhibit its rotation during securing the screws. After 10 minutes, the abutments were re-torqued to reduce the effect of embedment relaxation.^20^ Initial torque loss (ITL) values and ITL percentage were measured and recorded for each abutment in either group five minutes after the second screw tightening using an electronic torque wrench (Figure 3). Afterwards, retightening of the abutments was performed as described previously.

**Figure 4:**
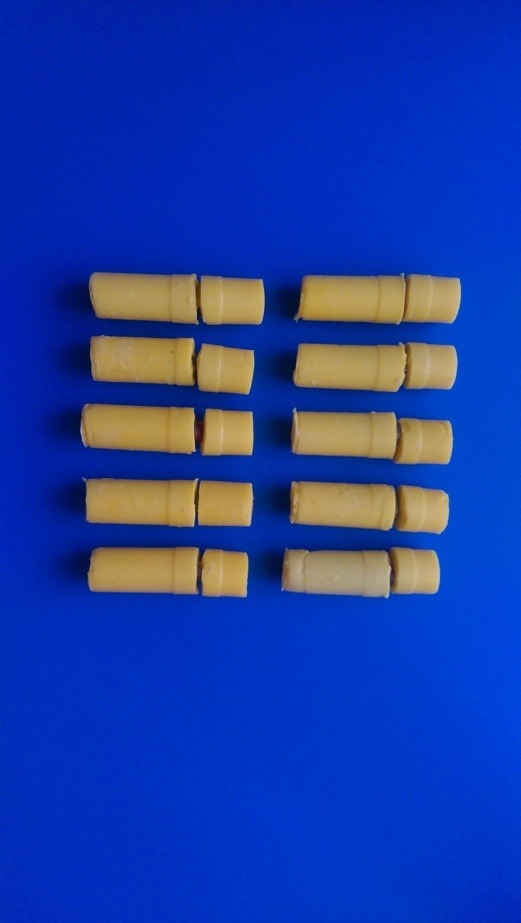
The implant‒abutment assemblies placed in putty to the interface in order to minimize the incident of bonding of radiotracer to their external surfaces.

The implant‒abutment (I/A) assemblies were placed in fast-setting putty (Panasil Putty, Kettenbach GmbH & Co. KG. Germany) to the interface of the I/A in order to minimize the incidence of bonding of radiotracer to their external surfaces (Figure 4). Cyanoacrylate adhesive was used in the interface for prevention of penetration of radiotracer between the putty and fixture. The positive control sample was not covered by putty, while the negative control sample was completely covered by putty.

The microleakage test was performed in two respective phases. In the first phase, the samples were placed into the thallium chloride-201 radiotracer solution of 0.5 mCi (milli Curie) in 100 mL of water for 24 hours before cyclic loading. Then the samples were retrieved from the radiotracer solution, cleansed with a detergent solution for 1 minute, followed by rinsing with distilled water; then the putty was removed and the samples were left to dry. Thereafter, the samples were placed into specially designed test tubes in the same position as each other. To count the photons in terms of count per minute (CPM), a gamma counter (Kontron, Gammamatic, Switzerland) with photo pick adjustment for thallium-201 (77 kev) and an energy window of 15% was employed for one minute.^21^ To remove the resultant radioactive contamination, the samples were quarantined in a lead-lined container for 12 days.

In order to make a template for all the samples, a burnout cylinder was placed on an abutment and waxed with 0.7-mm thickness in all the areas, measured with a digital caliper (Mitutoyo America Corp, Aurora, Ill). The occlusal plane was created with 45° of inclination.^22^ After casting this pattern, a silicone mold was made using polyvinyl siloxane impression material (Rapid, Coltene AG, Altstatten, Switzerland) to be used for all the samples. The patterns were invested using a phosphate-bonded investment (Cera-Fina, Whip Mix Corp, Louisville, Ky) and cast in base-metal alloy (Verabond 2, Albadent, Cordelia, Calif). After divesting the castings using aluminum oxide air abrasion, the inner irregularities were removed with a carbide bur (#169L-009; Brasseler Inc., Savannah, Ga). Silicone disclosing medium (Fit Checker, GC Corp, Tokyo, Japan) was also used to achieve the best fit.

Thereafter, to ensure easy removal of the crowns after cyclic loading, the crowns were seated on the abutments without any cement. Acrylic resin blocks were firmly mounted in a holder of a chewing simulator machine (Chewing Simulator, S-D Mechatronic, Germany) with a contact time of 0.2 seconds between the rod and crown with a frequency of 1 Hz.^23^ The cyclic loading test was performed with a force of 50 N perpendicular to the occlusal surface with 500000 cycles.^24^ After cyclic loading, the samples were removed from the acrylic block and the preparation procedures were performed as the first stage. Then, the second phase of microleakage test was carried out as mentioned before. Data from the microleakage tests were achieved before and after cyclic loading. Also, the removal torque value of each abutment was measured, and the percentage of secondary torque loss (STL) was calculated for each group (STL%).

To describe the quantitative variables the means ± SD were presented in Tables 1 and 2. In addition, because of the small sample size within each group, non-parametric tests were used (Wilcoxon test for comparing two related samples and Mann-Whitney test for comparing two independent samples.). P-values <0.05 were considered statistically significant.

## Results

The initial and secondary values of TL (torque loss) and PTL (percentage of torque loss) in each group are presented in Table 1. In addition, the mean differences of TL and PTL were compared between these two groups. According to the results, there was a significant difference between the two groups in terms of torque loss percentage (P=0.008), with the Noble Active group exhibiting a lower torque loss percentage than the Branemark group (9.8% and 39.8%, respectively) (Table 1). Also, the amount and percentage of torque loss after cyclic loading were increased within both groups. The Branemark group showed almost four times more torque loss after cyclic loading as compared to the Noble Active group (P=0.008) (Table 1).

**Table 1:** Comparison of the initial and secondary torque loss (ITL, STL) and percent of torque loss (PTL) within and between two groups

**Variable**	**Group**	**ITL**	**STL**	**P-value** ^*^	**Mean dif**	**P-value** ^**^
**TL**	**Branemark (External)**	7.40±1.82	21.40±22.70	0.042	14.00±2.35	0.008
	**Nobel Active (internal)**	8.60±2.07	12.00±3.67	0.042	3.40±1.82	
**PTL**	**Branemark (External)**	21.20±5.45	61.00±7.78	0.042	39.80±6.83	0.008
	**Nobel Active (internal)**	24.40±5.90	34.20±10.62	0.043	9.80±5.49	

*From Wilcoxon test for comparing ITL and STL values within each group. **From Mann-Whitney test for comparing mean differences between the two groups.

Two study groups exhibited an increase in gamma count after cyclic loading within each group. However, it was not significant within each group (Branemark, P=0.893; and Noble Active, P=0.225) (Table 2). Although the gamma count for the Branemark group was greater than the Noble Active group, the results indicated that the microleakage difference between the two groups was not significant after cyclic loading (P>0.841) (Table 2).

**Table 2:** Comparison of microleakage before and after cyclic loading within and between two groups

**Group**	**Before CL**	**After CL**	**P-value** ^*^	**Mean dif**	**P-value** ^**^
**Branemark (External)**	13657.60±12796.95	22004.00±25352.06	0.893	8346.40±31360.29	0.841
**Nobel Active (internal)**	16045.60±6607.33	26337.40±16330.67	0.225	10291.80±19108.17	

*From Wilcoxon test for comparing gamma count before and after cyclic loading within each group. **From Mann-Whitney test for comparing mean differences between the two groups.

## Discussion

The aim of the present study was to evaluate and compare microleakage and screw loosening of two different implant connections before and after cyclic loading. The null hypothesis regarding microleakage was supported since there was no significant difference between and within the two groups in terms of microleakage after cyclic loading. However, the null hypothesis about screw loosening was rejected due to significant difference in torque loss between and within the two groups after cyclic loading. Since microleakage would increase when the abutment is not torqued according to the recommended torque,^18^ all the abutments were tightened to the manufacturer-recommended torque in the present study.

External connection design was the first design in dental implants with the aim to simplify surgical placement and also provide an anti-rotational feature.^25^ Despite the advantages of external hexagon connection, there is an increased potential for screw loosening and fracture in this connection design.^26^ More screw loosening potential is consistent with the findings of this study. According to Maeda et al,^26^ as compared to external connection, internal hexagon interface is a more stable joint, especially for single-tooth restorations, and is more resistant to lateral loading. This statement is also consistent with our findings. The reason for such a result is probably the lower level of rotational center and more favorable stress distribution in the internal connection design under loading.^26^ The results also showed more torque loss percentage in the Branemark group. This finding is in agreement with other studies that indicated higher joint stability in internal conical connection designs as compared to external butt joints.^27,28^ According to Sakaguchi et al,^29^ when the same amount of tightening torque was applied to internal and external connections, the generated compressive force was higher in external connection. However, it has been reported that the stress created in the external connection is greater than that in the internal connection.^1^ This difference is attributed to the wedge effect in the internal connection due to the conical abutment sinking.^1^

Another finding of this study was a higher secondary torque loss in both groups as compared to initial torque loss before cyclic loading. This finding is consistent with most of other similar studies. However, few studies have shown that depending on the connection design, there may be an increase in the torque value after mechanical loading.^30,31^ The suggested reason for this finding has been the decrease in the gap at the abutment‒implant joint following the increased contact between their inner walls after cyclic loading in conical Morse taper connections.^31,32^ It should also be mentioned that eccentric loading would not necessarily lead to more torque loss as compared to centric loading.^30^

However, according to Kim et al^33^ despite statistically significant decrease in RTVs after loading in the internal hexagon and octagon groups with two-piece abutments, there was no significant differences in the external group, and also internal hexagon and octagon groups with one-piece abutments. Another study showed decreased RTVs in all the external and internal groups after cyclic loading.^34^ In addition, more loose screws were reported for externally connected implant systems as compared to internal ones.^25^ However, according to Tsuge and Hagiwara implant‒abutment connection geometry has no effect on screw loosening, and screw material and provision of proper anti-rotational features and tightening torque are more important.^35^

Although the bacterial counts after cyclic loading increased in each group, there was no significant difference between and within the two groups in terms of microleakage. A reason for this finding and also relative high SDs could be the relative low number of samples in each group. The literature is controversial on the effect of implant connection geometry on bacterial infiltration. Some studies have reported external connection as more prone to leakage than others.^15,17,37^ Furthermore, there are data supporting conical Morse taper internal connection as the lowest permeable to fluid leakage.^38,39^ This finding has been attributed to decreasing of the interface gap, especially after cyclic loading, which indicates better adaptation of the contacting surfaces.^32^ This explanation could also describe less crestal bone resorption associated with this connection.^36^ Furthermore, in comparison to Morse taper connection, external- and internal-hexagon implants have shown higher bacterial accumulation after mechanical loading.^13,36^ However, some authors have reported that internal conical joint are not completely safe in relation to microleakage.^12,15,18^ There are also articles that advocate that connection design and type has no influence on the bacterial leakage results.^17^ It is also known that there is a tendency for increasing the gap size under mechanical loading^7,12^ which is consistent with the results of this study.

Different methods could be used in order to detect microleakage the abutment‒implant interface such as bacterial incubation, chemical tracers, electerochemical changes, autoradiographic studies, electronic microscope, DNA checkerboard, gas-enhanced permeation test (GEPT), and dye infiltration (such as toluidine blue).^16,17,37^ Therefore, a reason for inconsistent findings on microleakage of different implant connections could be related to using different methods by different investigators. Radioisotope materials or radio-tracing could be used for detecting micro-gaps. The advantage of nondestructive radioisotope material which was used in the present study is its quantitative and reproducible nature.^41^ Tracer activity would be measured by a count of x rays emitted from the penetrated material into the implant body using a gamma camera/counter.^42^ Therefore, use of this method was one of the advantages of this study. However, further studies are necessary to evaluate other implant systems under centric and eccentric loading.

## Conclusion

Within the limitations of this study, the difference between the two connection types was significant in terms of torque loss, and the internal connection exhibited better torque maintenance compared to the external hexagon connection. In addition, the reverse torque values decreased in both group after cyclic loading. Furthermore, no significant difference was found between the external and internal connection types in terms of microleakage using radiotracing technique. The connection type proved not to be a factor in bacterial leakage after cyclic loading.

## Acknowledgments

The authors express special thanks to Hengam Dandan Companies for their generous support.

## Authors’ contributions

All authors have contributed to the concept and design of the study. BF supervised the conduct of the experiment. HS contributed to the data collection. HN, MA, BF, EB, FZ and HS contributed to the data analysis. HF and BF drafted the manuscript. All authors have read and approved the final paper.

## Competing interests

The authors declare that they have no competing interests with regards to authorship and/or publication of this work.

## Funding

This project was funded by a grant (# 15025) from Dental Implant Research Center, Tehran University of Medical Sciences.

## Ethics approval

Not applicable.
